# Type I Interferon Induction Is Detrimental during Infection with the Whipple's Disease Bacterium, *Tropheryma whipplei*

**DOI:** 10.1371/journal.ppat.1000722

**Published:** 2010-01-15

**Authors:** Khatoun Al Moussawi, Eric Ghigo, Ulrich Kalinke, Lena Alexopoulou, Jean-Louis Mege, Benoit Desnues

**Affiliations:** 1 Université de la Méditerranée, Centre National de la Recherche Scientifique, Unité Mixte de Recherche 6236, Marseille, France; 2 Paul-Ehrlich-Institut, Langen, Germany; 3 Center of Immunology Marseille-Luminy, Centre National de la Recherche Scientifique, Unité Mixte de Recherche 6102, Institut National de la Santé et de la Recherche Médicale U631, Université de la Méditerranée Unité Mixte de Recherche 6546, Campus of Luminy, Case 906, Marseille, France; Stanford University School of Medicine, United States of America

## Abstract

Macrophages are the first line of defense against pathogens. Upon infection macrophages usually produce high levels of proinflammatory mediators. However, macrophages can undergo an alternate polarization leading to a permissive state. In assessing global macrophage responses to the bacterial agent of Whipple's disease, *Tropheryma whipplei*, we found that *T. whipplei* induced M2 macrophage polarization which was compatible with bacterial replication. Surprisingly, this M2 polarization of infected macrophages was associated with apoptosis induction and a functional type I interferon (IFN) response, through IRF3 activation and STAT1 phosphorylation. Using macrophages from mice deficient for the type I IFN receptor, we found that this type I IFN response was required for *T. whipplei*-induced macrophage apoptosis in a JNK-dependent manner and was associated with the intracellular replication of *T. whipplei* independently of JNK. This study underscores the role of macrophage polarization in host responses and highlights the detrimental role of type I IFN during *T. whipplei* infection.

## Author Summary

Innate immune cells are sentinels allowing the host to sense invading pathogens. Among them, macrophages are highly microbicidal and are able to kill microorganisms. However, several pathogens have evolved strategies to hijack macrophage responses in order to survive or replicate. *Tropheryma whipplei* is the agent of Whipple's disease, a systemic disease that associates arthropathy, weight loss and gastrointestinal symptoms. It has been known for several years that this bacterium has a tropism for macrophages, in which it replicates. In this study, we have shown that *T. whipplei* induces host cell apoptosis and a surprising macrophage activation, characterized by anti-inflammatory molecules and type I interferon (IFN) signaling, which is generally associated to viral infections. We demonstrate that this type I IFN response is critical for bacterial pathogenicity, as it is required for bacterial replication and provides the first step of the apoptotic program of infected macrophages. By identifying these signaling events induced in macrophage by *T. whipplei*, we can now better understand the molecular basis of the pathophysiology of Whipple's disease, of interest for clinical and therapeutic ends.

## Introduction

Over the past decades, activated macrophages were mainly considered as cells that secrete inflammatory mediators and kill intracellular pathogens. However, studies have now revealed activated macrophages as a continuum of cells with phenotypic and functional heterogeneity [1],[2]. Schematically, macrophages exposed to the classic activation signals (lipopolysaccharide (LPS) and/or IFN-γ) polarize into the M1 phenotype and express high levels of TNF, IL-1, IL-6, IL-12, type I IFN, inflammatory chemokines, such as CXCL10, and inducible nitric oxide synthase. In contrast, M2 macrophages, induced by IL-4, IL-10 or immune complexes, are characterized by the expression of non-opsonic receptors, arginase, and the absence of proinflammatory cytokines [3],[4]. Recently, we defined a “common host response” of macrophages to bacterial infections, characterized with an M1 signature and associated with the control of acute infections. However, successful infection by pathogenic intracellular bacteria usually relies on the perturbation or avoidance of the classical M1 proinflammatory activation profile [3].

Recognition of microorganisms by macrophages is mediated by pattern recognition receptors (PRR) that bind conserved microbe-associated molecular patterns (MAMPs) [5]. PRR engagement by MAMPs activates a major signaling cascade that leads ultimately to the activation of mitogen-activated protein (MAP) kinases and the transcription factors NF-κB and IRF3 [5],[6]. These transcription factors then migrate to the nucleus where they drive the transcription of proinflammatory genes and type I IFN genes, respectively [7]. Type I IFN are responsible for inducing transcription of a subset of genes referred as interferon stimulated genes. Classically, type I IFN transcription is first activated by signals that induce cooperative binding of the transcription factors c-Jun/ATF2, NF-κB and interferon regulatory factor-3 (IRF3) to the promoter [8]. Following stimulation with viral or bacterial components, the constitutively expressed IRF3 is phosphorylated in the cytoplasm, dimerizes and then translocates in the nucleus to induce the transcription of type I IFN [9]. Once secreted, type I IFN initiates a positive feed-back loop through binding to its receptor IFNAR [8]. IFNAR activates the protein tyrosine kinases JAK1 and JAK2 which phosphorylate STAT1 and STAT2 to further drive the transcription of a large group of IFN inducible genes [10].

Stimulation with Gram-negative bacteria or LPS induces type I IFN, at least partially through Toll-like receptor (TLR) 4 [11]. In addition, the intracellular pathogens *Shigella flexnerii*, *Legionella pneumophila* and *Francisella tularensis* induce a potent type I IFN response while non invasive mutants do not [12]–[14]. MAMPs from Gram-positive bacteria are also able to induce type I IFN. Indeed, *Listeria monocytogenes* triggers type I IFN, probably through bacterial DNA recognition by a cytosolic receptor [15],[16]. Infection of various cell types with *Mycobacterium tuberculosis* has also been shown to induce type I IFN [17]. Recently, the extracellular pathogen group B Streptococcus has been shown to induce type I IFN in a TLR-independent manner through intracellular recognition of its DNA [18]. Remarkably, stimulation of macrophages with most of these bacteria and/or bacterial ligands induces M1 polarization, strongly supporting the fact that type I IFN response is a feature of classical activation of macrophages. This point is strengthened by the fact that type I IFN significantly contribute to the cross-talk between the MyD88-dependent and MyD88-independent pathways, enabling full responsiveness to LPS [19].

Here, we have studied and characterized mouse macrophage responses to infection with the facultative intracellular Gram positive bacterium *Tropheryma whipplei*, the etiologic agent of Whipple's disease [20]. Whipple's disease is a rare systemic disease that associates arthropathy, weight loss and gastrointestinal symptoms [21] but its pathophysiology remains largely unknown. Recently, we provided major insights into the understanding of host immune factors in Whipple's disease, delineating macrophage polarization and apoptosis as critical in the pathophysiology of the disease [3], [22]–[24]. In this report, we provide evidence that, besides M2 macrophage polarization and apoptosis, *T. whipplei* induced a robust type I IFN response. This response required bacterial viability and was associated with bacterial intracellular replication. We also observed that *T. whipplei* induced macrophage apoptosis in a type I IFN- and JNK- dependent manner. These findings reveal an unexpected type I IFN response associated with M2 polarization.

## Results

### Transcriptional program induced by *T. whipplei*

**Figure 1:**
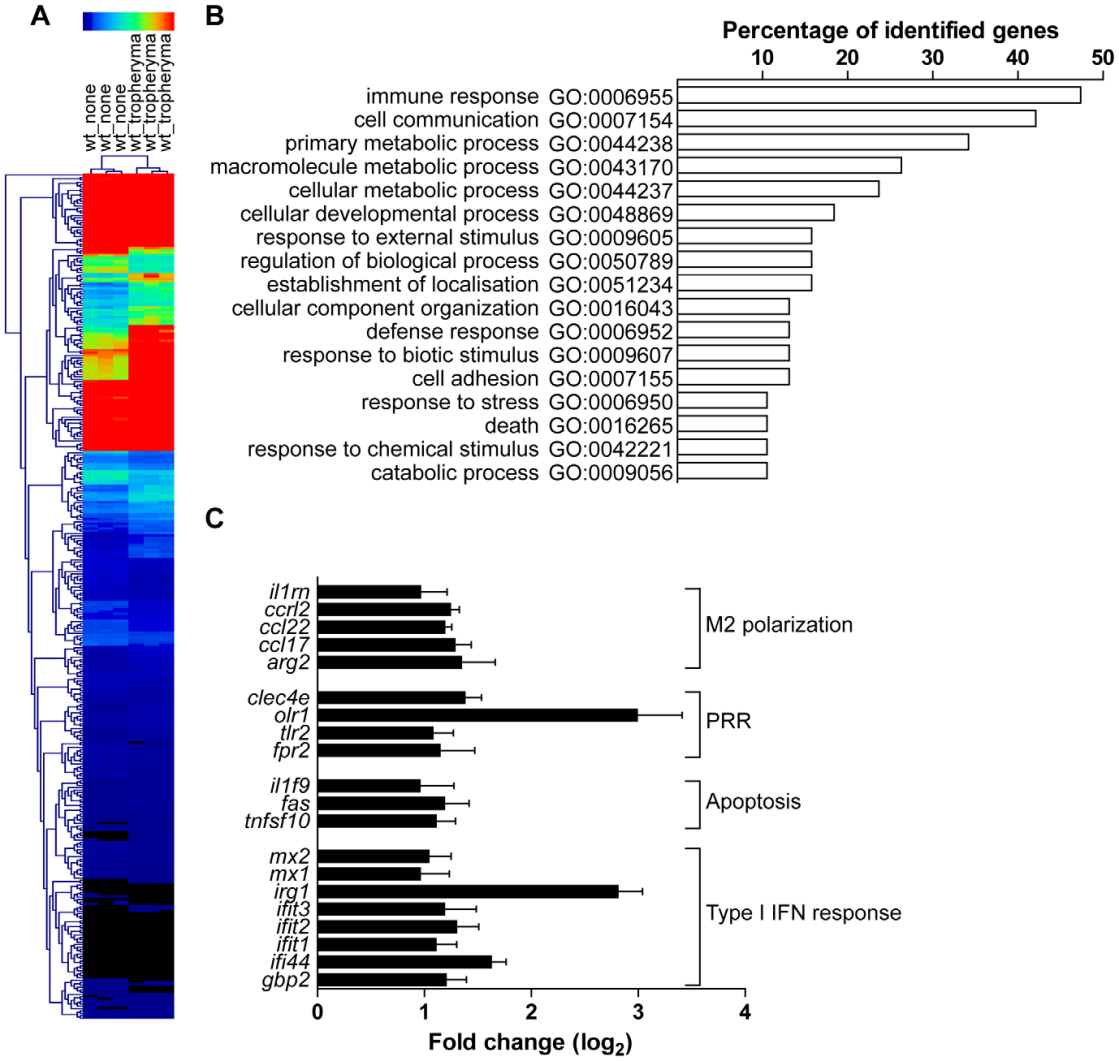
Transcriptional program induced *T. whipplei* in BMDM. BMDM were stimulated with *T. whipplei* (MOI 50∶1) for 6 h and transcriptional responses were monitored by microarrays. (A) Gene expression values were normalized by trimmed means followed by the unpaired Student's *t* test (P<0.01) and agglomerative hierarchical clustering of 356 spotted features was based on expression patterns between samples. Rows colorimetrically represent expression values of individual genes from black (low expression) to red (high expression). (B) *T. whipplei*-regulated genes were assigned functional categories by determining genes statistically over represented through Gene Ontology (GO) annotations. The diagram displays all statistically relevant GO groups among the up-regulated genes. (C) Immune response GO group-belonging genes were then classified in functional sub-categories after dividing gene expression values from *T. whipplei*-stimulated BMDM by expression values from mock-treated values to derive fold change ratios.

To evaluate gene expression profiles, bone marrow-derived macrophages (BMDM) were infected with *T. whipplei* for 6 h and transcriptional response was examined by microarray analysis. Of the 43,379 spotted features, 356 were significantly modulated in response to *T. whipplei* infection (P<0.01, Fig. 1A). To increase the reliability of our datasets, we considered transcripts as significantly regulated if they showed at least a 2-fold modulation in gene expression levels. We overall identified 59 and 11 genes that were up- and downregulated, respectively. Upregulated genes were assigned to biological process gene ontology (GO) categories. Around 50% of them belonged to the immune response GO group (Fig. 1B). These immune response genes could be sub-classified in 4 functional categories. In the first category were genes linked to macrophage polarization and more specifically to M2 polarization (Fig. 1C). Indeed, genes for the prototypal M2 markers interleukin 1 receptor antagonist (*il1rn*) and arginase 2 (*arg2*), as well as the M2 chemokines Ccl17 and Ccl22 were induced, while none of the M1 markers were modulated. The second set of the immune response-related genes represented genes related to PRR (Fig. 1C). In this group, 2 genes encoding lectins were markedly induced: *clec4e* which encodes a C-type lectin, and *olr1*, which encodes the lectin-like oxidized low density lipoprotein receptor 1. In addition, *T. whipplei* also upregulated the expression of *tlr2*, involved in the recognition of Gram-positive bacteria, and that of formyl peptide receptor 2, encoded by *fpr2*, which mediates the chemotactic activity of a variety of pathogen and host-derived peptides. The third group of immune response-associated genes included apoptosis-related genes. Indeed, we found that *fas*, and *tnfsf10* were efficiently induced in BMDM following stimulation with *T. whipplei* (Fig. 1C). Finally, we isolated a fourth set of immune response-related genes that contained genes involved in the type I IFN response (Fig. 1C). In this group were found the genes encoding Mx1 and Mx2, which mediate resistance against negative-strand RNA viruses, but also the IFN-stimulated genes *ifit1*, *ifit2* and *ifit3*, also known as *isg56*, *isg54* and *isg49*, respectively. Three other IFN-inducible genes (*irg1*, *ifi44* and *gbp2*) were among the most induced genes by *T. whipplei*. Selected genes were studied by quantitative real time RT-PCR. Upregulation of these exemplary genes was confirmed and statistical analysis revealed a significant correlation between microarray and RT-PCR data (Table 1).

**Table 1:** Validation of the microarray.

gene	microarray	qRT-PCR
*irg1*	6.7	7.1
*olr1*	6.7	2.9
*ifi44*	2. 9	2.2
*mmp14*	2.8	2.1
*arg2*	2.6	2.6
*ccl17*	2.3	3.2
*ccl22*	2.2	1.2
*ifit2*	2.1	2.0
*ifit1*	2.1	2.4
*mx2*	2.0	1.5
*nfkbie*	2.0	1.7
*mx1*	2.0	1.7

Microarray results were confirmed by qRT-PCR. Results are expressed as the ratio of expression levels in infected cells vs. uninfected cells. For qRT-PCR, ratio were calculated relative to beta actin control.

### *T. whipplei*-induced M2 polarization is related to a weak proinflammatory signaling and favors bacterial replication

The transcriptional program of BMDM elicited by *T. whipplei* revealed a marked polarization towards a M2 phenotype. This macrophage functional activation state is characterized by the absence of proinflammatory mediators [3]. We investigated the lack of proinflammatory response by examining the activation of the transcription factor NF-κB and the phosphorylation of MAPK in response to *T. whipplei*.

**Figure 2:**
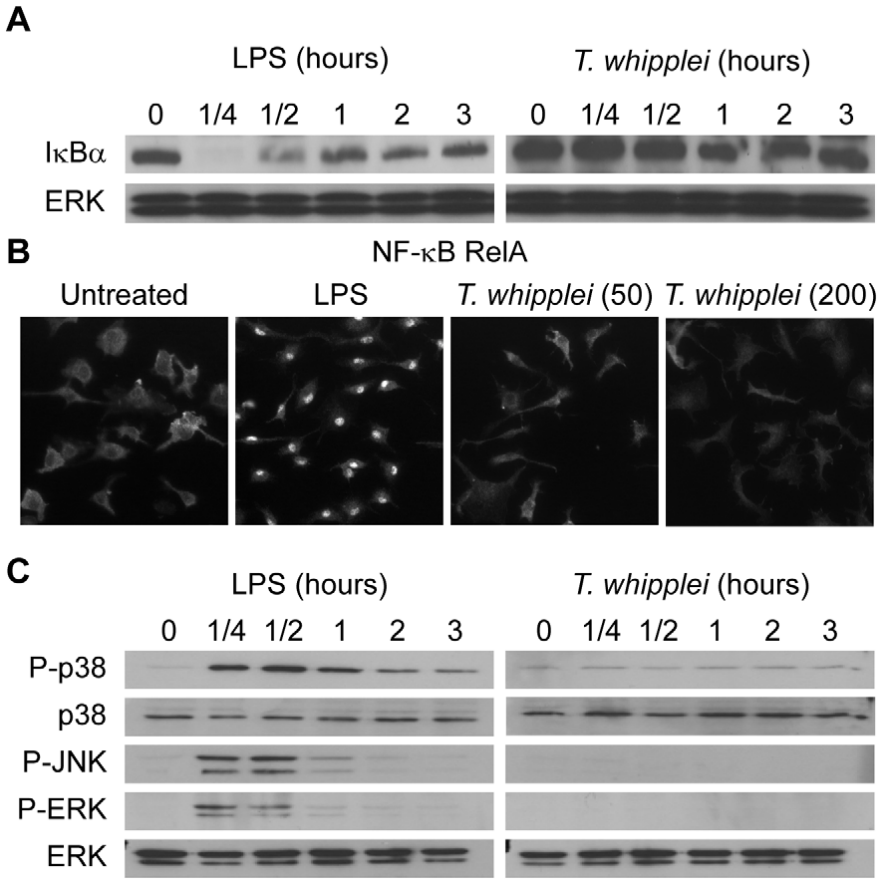
*T. whipplei* poorly induces NF-κB and MAPK pathways. (A) BMDM were stimulated with LPS (100 ng/ml) or *T. whipplei* (MOI 50∶1) for the indicated time points, and lysates were analyzed by immunoblotting. IκBα blots were stripped and reprobed for ERK as loading control. One representative experiment is shown (*n* = 3). (B) BMDM were stimulated for 1 h with LPS (100 ng/ml) or *T. whipplei* (MOI 50∶1 or 200∶1). RelA nuclear translocation was determined by immunofluorescence using p65-specific antibodies. (C) BMDM were stimulated with LPS (100 ng/ml) or *T. whipplei* (MOI 50∶1) for the indicated time points, and lysates were analyzed by immunoblotting. Phospho-p38, phospho-ERK and Phospho-JNK blots were stripped and reprobed for p38 and ERK as loading controls. One representative experiment is shown (*n* = 3).

NF-κB activation was assessed by determining changes in cytoplasmic IκBα protein levels. LPS (100 ng/ml) clearly induced NF-κB activation. Indeed, a transient degradation of IκBα, maximal at 15 min was observed (Fig. 2A). This profile was in agreement with kinetics of RelA translocation in the nucleus (Fig. 2B). In contrast, stimulation with *T. whipplei* induced a faint IκBα degradation between 1 h and 2 h and IκBα levels increased back normal by 3 h (Fig. 2A). However, RelA translocation was not observed, even by increasing 4 fold the dose of bacteria (Fig. 2B). Nevertheless, the fact that IκBα increased back to initial levels at 3 h suggest that *T. whipplei* is a weak activator of NF-κB.

Besides NF-κB, we assessed MAPK activation in response to *T. whipplei*. BMDM were stimulated with 100 ng/ml LPS or *T. whipplei* for 15 min to 3 h and, subsequently, analyzed for phosphorylation of the MAPKs, p38, Erk1/2, and JNK. In the first 15–30 min after LPS stimulation, transient phosphorylation of all kinases could be detected (Fig. 2C). In contrast, when BMDM were stimulated with *T. whipplei*, no phosphorylation of the MAPKs p38, ERK and JNK could be observed during the 3 hour-time-frame (Fig. 2C). Increasing the doses of *T. whipplei* had no effect on MAPK activation (data not shown).

**Figure 3:**
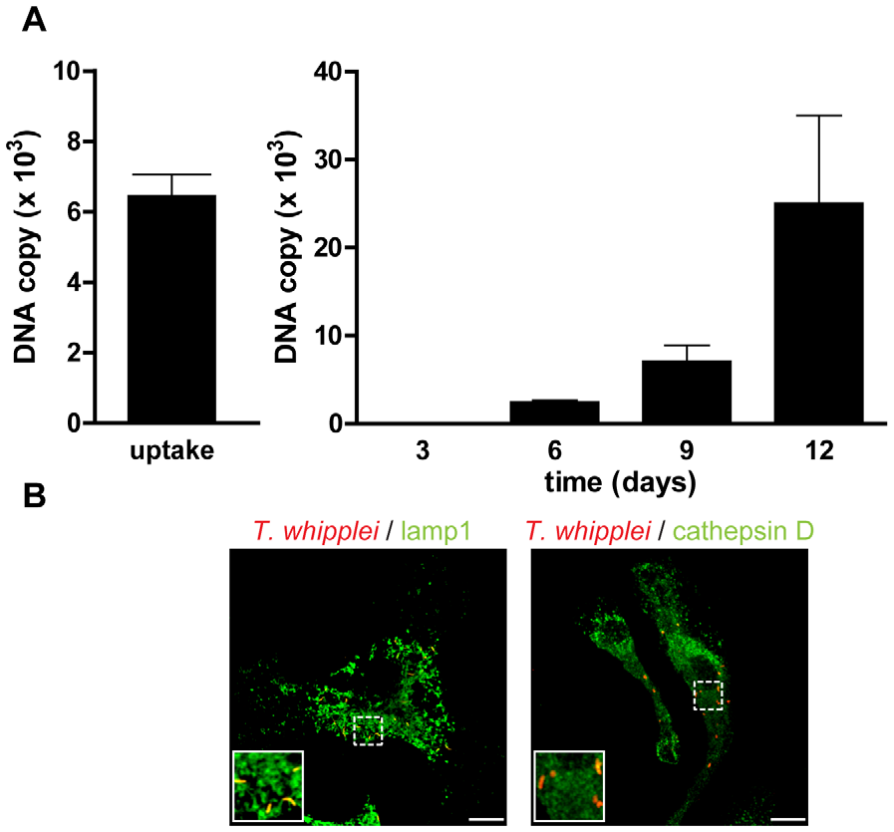
*T. whipplei* replicates in BMDM. (A) BMDM were infected with *T. whipplei* (MOI 50∶1) for 4 h (uptake), washed and incubated for different periods. Levels of bacterial DNA copy number were determined by qPCR (*n* = 3). (B) At day 12 post infection, *T. whipplei* organisms, lamp-1 and cathepsin D were visualized by laser scanning microscopy.

As BMDM were poorly proinflammatory, it is likely that they allowed *T. whipplei* replication. Therefore, BMDM were infected with *T. whipplei* and bacterial uptake and replication was assessed by qPCR. BMDM efficiently internalized *T. whipplei* as around 6,000 bacterial DNA copies were detected after 4 h of infection (Fig. 3A). In the first 3 days, bacterial DNA copy number decreased and started to increase after 6 days and reached around 30,000 copies after 12 days (Fig. 3A). These results were further investigated by examining the vacuole containing *T. whipplei* at day 12 post infection. The great majority of bacteria colocalized with the late phagosome marker lamp1 (92%±11%); however, these *T. whipplei*-containing vacuoles excluded the lysosomal hydrolase cathepsin D (20%±15%, Fig. 3B).

Overall, these results showed that *T. whipplei* infects BMDM, induces M2 polarization and replicates, at least by interfering with phagosome conversion.

### Functional type I IFN signalling induced by *T. whipplei*

Besides M2 polarization, BMDM response profiling to *T. whipplei* infection revealed a striking induction of type I IFN-inducible genes. Some genes, among which *ifnb1* and *cxcl10*, which encode respectively IFN-β and the chemokine Cxcl10, were excluded from the analysis when we applied our criterion; however, *ifnb1* was up-regulated 1.6 times and *cxcl10* 4.2 times. To further confirm type I IFN induction following *T. whipplei* infection, we performed time course experiments. Expression of IFN-β mRNA increased to reach a maximal level at 6 h after infection and then was shut off, as revealed by its low expression value at 24 h (Fig. S1A). Consistent with transcriptional data, IFN-β protein was secreted by infected BMDM at 3 h and reached maximal levels 6 h post infection (Fig. S1B), exemplifying the importance of the type I IFN pathway during *T. whipplei* infection.

**Figure 4:**
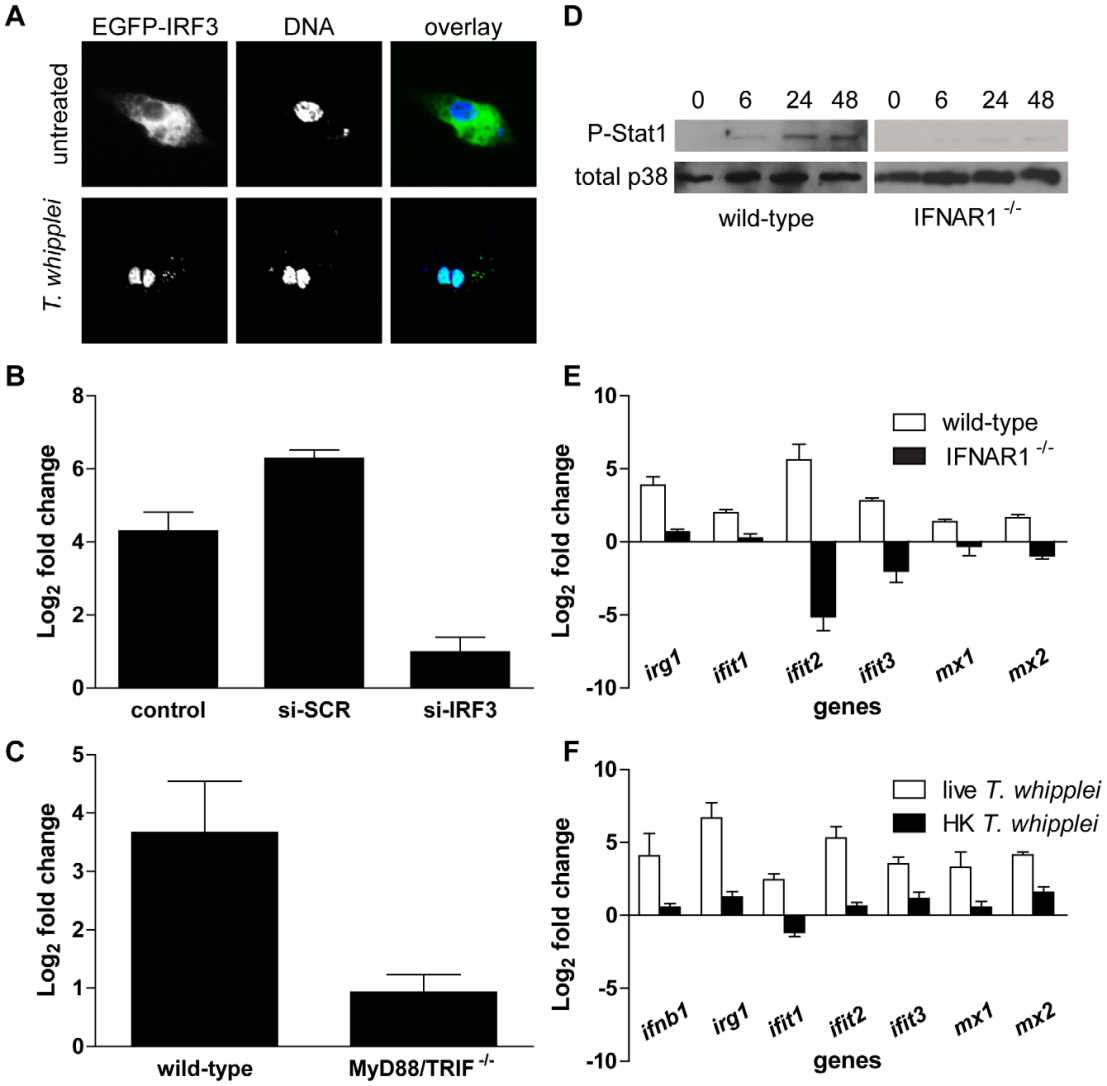
*T. whipplei* induces a functional type I IFN response. (A) RAW cells were transiently transfected with IRF3-EGFP before stimulation with *T. whipplei* (MOI 50∶1) for 4 h. Nuclei were stained with DAPI. (B) RAW cells were transiently transfected with IRF3-specific siRNA (si-IRF3), control scramble siRNA (si-SCR) or left untransfected (control) 24 h before stimulation with *T. whipplei* (MOI 50∶1). IFN-β expression was monitored after 6 h using qRT-PCR. Results are expressed as the ratio of expression levels in infected cells vs. uninfected cells relative to β actin). (C) BMDM from wild-type and MyD88/TRIF^−/−^ mice were stimulated with *T. whipplei* (MOI 50∶1) for 6 h and IFN-β expression was monitored using qRT-PCR. Results are expressed as the ratio of expression levels in infected cells vs. uninfected cells relative to β actin). (D) BMDM from wild-type and IFNAR1^−/−^ mice were stimulated for indicated time points with *T. whipplei* (MOI 50∶1), and lysates were analyzed by immunoblotting. Phospho-STAT1 blots were stripped and reprobed for p38 as loading control. One representative experiment is shown (*n* = 3). (E) BMDM from wild-type and IFNAR1^−/−^ mice were stimulated with *T. whipplei* (MOI 50∶1) for 6 h and host responses were monitored using qRT-PCR on indicated genes. Results are expressed as the ratio of expression levels in infected cells vs. uninfected cells relative to β actin). (F) BMDM were stimulated with live and heat-killed *T. whipplei* (MOI 50∶1) for 6 h and host responses were monitored by qRT-PCR on indicated genes. Results are expressed as the ratio of expression levels in infected cells vs uninfected cells relative to beta actin.

Induction of IFN-β is thought to depend on the constitutively expressed transcription factor IRF3 [9]. We therefore, determined the subcellular localization of IRF3 following *T. whipplei* stimulation. Raw 264.7 macrophages overexpressing EGFP-IRF3 were incubated with *T. whipplei* for 4 h. Confocal microscopy allowed to visualized a marked nuclear translocation of IRF3 in response to *T. whipplei* (Fig. 4A) while, in unstimulated cells, IRF3 remained in the cytosol. This result suggests that the type I IFN induction depends on the transcription factor IRF3. To further examine the role of IRF3 in type I IFN induction by *T. whipplei*, we used siRNA technology. Transfection of IRF3-specific siRNA (si-IRF3) in Raw 264.7 macrophages resulted in a dramatic reduction of IRF3 levels at 24 h (84%), as determined by Western blot, while control scramble siRNA (si-SCR) had no effect (Fig. S2A). IRF3-specific siRNA action was transient since IRF3 levels were back to normal 48 h post transfection. Therefore, we selected the 24 h time point to monitor the effect of IRF3 inhibition on IFN-β expression. Inhibition of IRF3 led to a profound reduction of IFN-β expression following *T. whipplei* stimulation, compared to control siRNA (Fig. 4B). As a result, IFN-β production was reduced by 94% in cells lacking IRF3 (Fig. S2B), thus identifying IRF3 as a component of the signalling pathway leading to type I IFN induction by *T. whipplei*-infected macrophages.

In order to understand how *T. whipplei* turns on the type I IFN response, we examined the potential contribution of TLRs, the signalling of which is known to ultimately involve the adaptor molecules MyD88 and/or TRIF [25]. BMDM from double MyD88 and TRIF-deficient (MyD88/TRIF^−/−^) mice, which are unable to respond to TLR agonists, were stimulated with *T. whipplei* and IFN-β expression was monitored by qRT-PCR. Results showed that in contrast to wt BMDM, IFN-β expression was abrogated in MyD88/TRIF^−/−^ BMDM (Fig. 4C), suggesting that TLR signalling is required for type I IFN response during *T. whipplei* infection.

Next, we wondered whether *T. whipplei* induces IFN-inducible genes via a type I IFN autocrine loop after engagement of the type I IFN receptor [26]. Thus, we first monitored the activation of the Stat1 transcription factor, one outcome of IFN secretion and type I IFN receptor engagement [27]. STAT1 activation was measured using specific antibodies targeting STAT1 phosphorylated at tyrosine 701. As shown in Figure 4D, an increase in Stat1 Tyr701 phosphorylation after *T. whipplei* stimulation was evidenced at 6 h with further elevation at 24 h and 48 h. In contrast, Stat1 phosphorylation was completely absent when BMDM knocked-out for the type I IFN receptor gene (IFNAR1^−/−^) were stimulated with *T. whipplei* (Fig. 4D).

Subsequently, we analyzed the *irg1*, *ifit1*, *ifit2*, *ifit3*, *mx1* and *mx2* gene induction following *T. whipplei* stimulation in IFNAR1^−/−^ BMDM. BMDM from wt and IFNAR1^−/−^ mice were stimulated for 6 h and RNA were subjected to qRT-PCR. As expected, *T. whipplei* induced a marked expression of these genes (Fig. 4E). However, the absence of type-I IFN receptor, which blocks the type I IFN autocrine induction, dramatically inhibited *irg1*, *ifit1*, *ifit2*, *ifit3*, *mx1* and *mx2* gene induction by *T. whipplei* (Fig. 4E). Finally, only live bacteria induced type I response, as heat-killed forms of *T. whipplei* did not induce transcription of *ifnb1*, *irg1*, *ifit1*, *ifit2*, *ifit3*, *mx1* and *mx2* (Fig. 4F).

Overall, these results indicate that type I IFN response is induced by viable *T. whipplei* organisms and likely involves a type I IFN autocrine loop.

### Type I IFN-dependent MAPK signalling in *T. whipplei* infected BMDM

**Figure 5:**
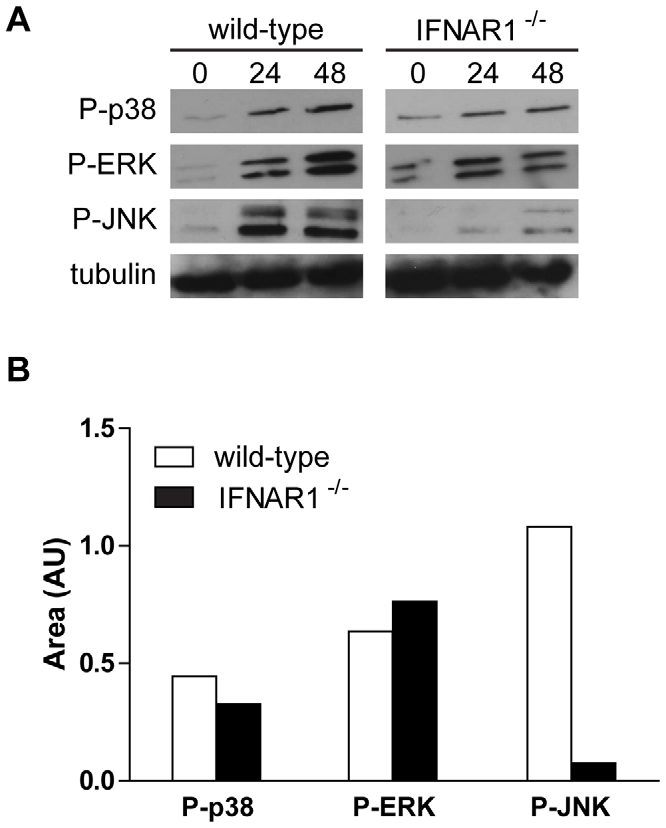
Late MAPK signaling partly depends on type I IFN receptor. (A) BMDM from wild-type and IFNAR1^−/−^ mice were stimulated with *T. whipplei* (MOI 50∶1) for indicated time points, and lysates were analyzed by immunoblotting. Phospho-p38, phospho-ERK and phospho-JNK blots were stripped and reprobed for tubulin as loading controls. One representative experiment is shown (*n* = 3). (B) Densitometry values of the phospho-p38, phospho-ERK and phospho-JNK autoradiographs were normalized to tubulin.

As members of the MAPK family are activated following the engagement of the type I IFN receptor and participate in the generation of IFN signals [28], we treated BMDM with *T. whipplei* and MAPK activation was followed through their phosphorylation state at 24 h and 48 h. Interestingly, we found that p38, ERK and JNK were phosphorylated at 24 h and their phosphorylation remained detectable 48 h after *T. whipplei* infection (Fig. 5A). In order to examine whether this late MAPK induction was attributable to type I IFN signalling, we monitored the activation of p38, ERK and JNK in IFNAR1^−/−^ BMDM. After stimulation with *T. whipplei*, p38 and ERK activities were increased at 24 h and were still detectable at 48 h (Fig. 5A). Conversely, the immunoreactive band of phospho-JNK was poorly if not detected in IFNAR1^−/−^ BMDM stimulated for 24 h and 48 h (Fig. 5A). Densitometry of the phosphorylated p38, ERK and JNK 24-h autoradiographs confirmed the differences in band intensity (Fig. 5B). These results suggest that *T. whipplei* induces a late MAPK signalling, which is, at least for JNK, dependent on the type I IFN receptor engagement.

### Type I IFN signalling sensitizes BMDM to cell death following *T. whipplei* infection

**Figure 6:**
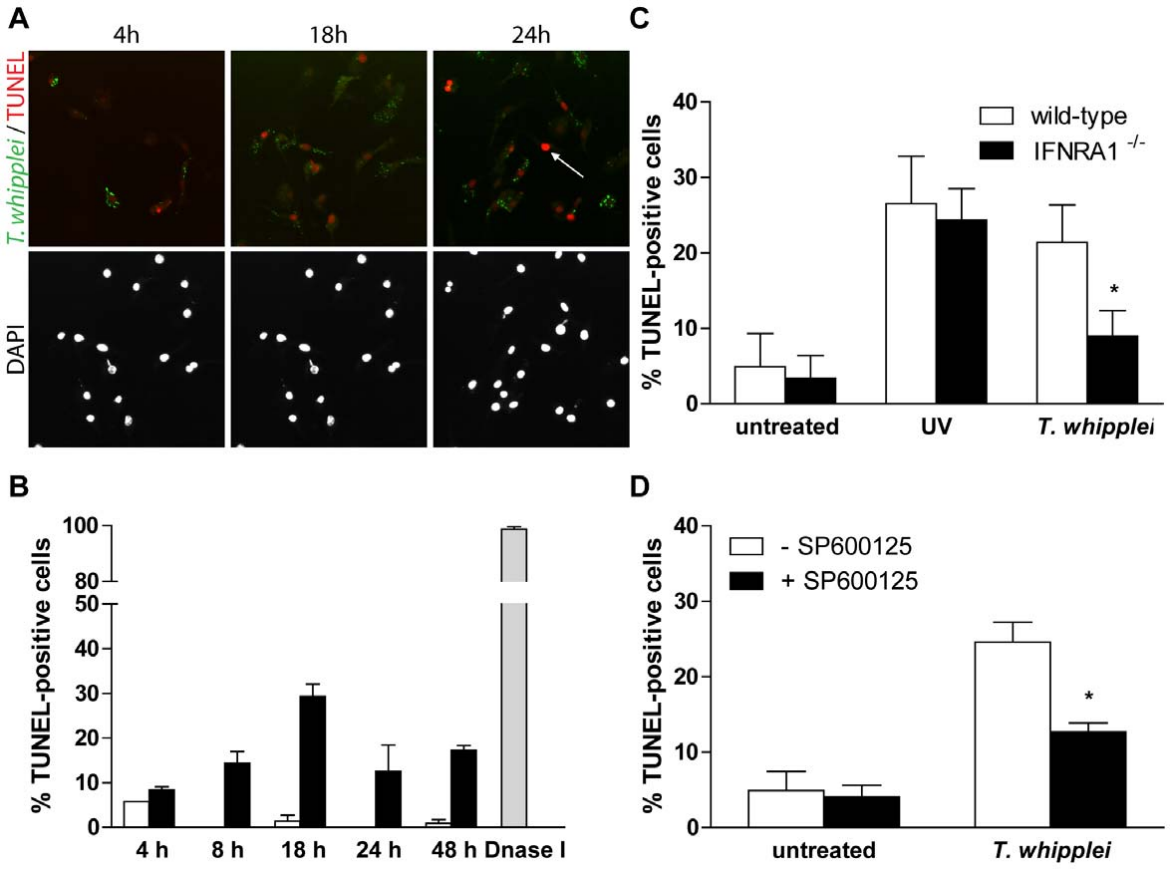
*T. whipplei*-induced apoptosis depends on type I IFN through JNK activity. (A) BMDM were infected with *T. whipplei* (MOI 50∶1) for 4 h and incubated for the indicated times. Apoptotic cells were revealed by TUNEL reaction, while *T. whipplei* organisms were revealed using a *T. whipplei* specific antibody. Nuclei were stained with DAPI. (B) Apoptosis was quantified by examining 3 to 5 fields per condition (100 to 300 cells each). The percentage of TUNEL-positive cells was calculated as the ratio between TUNEL-positive and DAPI-stained nuclei ×100 (white bars: uninfected cells, black bars: *T. whipplei*-infected cells, gray bar: DNaseI-treated cells). (C) BMDM from wild-type and IFNAR1^−/−^ mice were infected with *T. whipplei* (MOI 50∶1) for 4 h and incubated for 18 h. As a control, cells were exposed to UV. Apoptosis was determined as the percentage of TUNEL-positive cells. (*, P<0.05). (D) BMDM were treated or not with the JNK specific inhibitor SP600125 for 30 min. Cells were then infected with *T. whipplei* (MOI 50∶1) for 4 h and incubated for 18 h. Apoptosis was determined as the percentage of TUNEL-positive cells. (*, P<0.05).

Type I IFNs are known to induce apoptosis, at least in part through up-regulation of tumor necrosis factor (TNF) family proteins such as CD95 (Fas) [29]. In addition, we previously showed that *T. whipplei* induces apoptosis of human macrophages and that circulating apoptotic markers are increased during active Whipple's disease [22],[23]. To explore whether *T. whipplei* induces apoptosis of BMDM and whether type I IFN was involved, we measured BMDM apoptosis by TUNEL assay in time course experiments. We observed a gradual increase of TUNEL-positive cells that peaked at 18 h post infection, with nearly 25% of apoptotic cells (Fig. 6A and 6B). Thereafter, the number of apoptotic cells slightly decreased and remained stable at 15%, 48 h post infection (Fig. 6B). Interestingly, double-labeling of the apoptotic nuclei and *T. whipplei* in BMDM revealed that apoptosis was induced in infected cells, but also in cells that had not engulfed bacteria (Fig. 6A, arrow).

In IFNAR1^−/−^ BMDM, *T. whipplei*-induced apoptosis at 18 h was significantly reduced as compared to wt BMDM (Fig. 6C). This was not due to delayed apoptosis since incubating cells for longer periods did not reveal significant changes in cell death (data not shown). In addition, UV exposure of IFNAR1^−/−^ BMDM induced cell apoptosis at a level comparable to that of UV-treated wt BMDM (Fig. 6C), ruling out the fact that the IFN receptor would have been required for apoptosis induction.

Some studies have demonstrated that JNK plays a pivotal role in the activation of the apoptotic pathways [30]. As JNK was not activated and apoptosis was significantly reduced in *T. whipplei*-infected IFNAR1^−/−^ BMDM (see Fig. 5A and 6C), we wondered if JNK was required for *T. whipplei*-induced BMDM apoptosis. BMDM from wt mice were treated with the JNK specific inhibitor SP600125 for 30 min prior *T. whipplei* infection and apoptosis was measured after 18 h. We found that JNK inhibition significantly prevented *T. whipplei*-induced apoptosis (Fig. 6D). Taken together, these results confirm that the transcriptional proapoptotic pattern induced by *T. whipplei* is functional and indicate that *T. whipplei*-induced apoptosis is dependent on an autocrine/paracrine loop involving type I IFN, its receptor IFNAR1 which leads to JNK activation.

### Type I IFN is associated with *T. whipplei* replication

**Figure 7:**
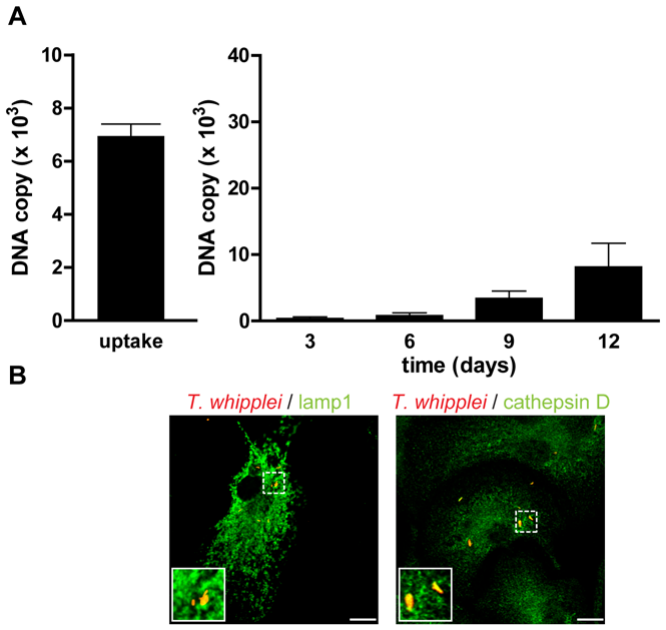
*T. whipplei* replication is impaired in IFNAR1^−/−^ BMDM. (A) BMDM from IFNAR1^−/−^ mice were infected with *T. whipplei* (MOI 50∶1) for 4 h (uptake), washed and incubated for different periods. Levels of bacterial DNA copy number were determined by qPCR (*n* = 3). (B) At day 12 post infection, *T. whipplei* organisms, Lamp-1 and cathepsin D were visualized by laser scanning microscopy.

Puzzled by these findings, we wondered if bacterial replication was linked to type I IFN signaling. Thus, we infected BMDM from IFNAR1^−/−^ mice with *T. whipplei* for 4 h and assessed bacterial replication. As expected, results showed that the type I IFN receptor was not involved in bacterial uptake, as around 7,000 bacterial DNA copies were detected after 4 h infection (Fig. 7A), which were comparable to that found in wt BMDM (Fig. 3A). However, replication of *T. whipplei* was reduced in these BMDM: around 10,000 bacterial DNA copies were detected at day 12 (Fig. 7A, compare with Fig. 3A, 30,000 copies at day 12), suggesting that type I IFN-dependent signaling is involved in macrophage permissivity to *T. whipplei*. Interestingly, we found that the killing of *T. whipplei* in IFNAR1^−/−^ BMDM was associated with the maturation of *T. whipplei*-containing phagosomes, as *T. whipplei* colocalized with both Lamp1 and cathepsin D (85%±14% and 97%±5%, respectively) at day 12 (Fig. 7B). Finally, we wondered if type I IFN, JNK activation and bacterial replication were related. Hence, BMDM from wt mice were treated with a JNK-specific inhibitor. Bacterial survival and the nature of *T. whipplei*-containing phagosome were monitored by qPCR and confocal microscopy, respectively. JNK inhibition revealed cellular toxicity beginning at day 6. However, during the first 6 days, bacterial replication was similar in untreated and SP600125-treated BMDM (Fig. S3A). In addition, most bacteria colocalized with lamp1 (83%±13%), but not with cathepsin D (5%±3%) at day 6 in both untreated and SP600125-treated BMDM (Fig. S3B). Overall, these results showed that *T. whipplei*-induced type I IFN response governs bacterial replication through modulation of the phagosome conversion, independently of JNK activation.

## Discussion

A key requirement for dissecting the complex role of macrophages during infection is to understand how microbes activate or regulate host cells. In this study, we examined and characterized host responses induced by the facultative intracellular pathogen *T. whipplei*.

Using microarray analysis of bone marrow-derived macrophages, we identified 59 genes that were significantly up-regulated upon infection. By bioinformatical approach, we found that most prominent GO groups covered immune response and cell communication. A closer analysis revealed that these over-represented genes could be classified in 4 functional categories. First, we found that *T. whipplei* induced M2 polarization of BMDM, which is consistent with the transcriptional profile of intestinal infiltrating cells, mainly comprised of macrophages, from patients with Whipple's disease [24]. Arginase, the M2 chemokines Ccl22 and Ccl17, and the IL-1 receptor antagonist were induced in macrophage following infection as it has been described in Whipple's disease lesions [24]. M2 macrophages differ from classically activated M1 macrophages in terms of receptors, cytokine/chemokine expression, and effector functions. As a result, while M1 macrophages are microbicidal and inflammatory, M2 macrophages are rather seen as immunomodulators with diminished microbicidal activities [3]. We show that *T. whipplei* was able to invade and replicate within BMDM in a similar fashion to that observed in human macrophages [22], suggesting that i) mouse macrophages constitute model cells to study *T. whipplei* – macrophage interaction and ii) *T. whipplei*-induced M2 polarization is a general response to this pathogen. Macrophage PRR responsible for *T. whipplei* recognition are still unknown. However, TLR2 and FPR2, which encodes the mouse homolog formyl peptide receptor 2 of the human G-protein-coupled formyl peptide like receptor 1 were upregulated upon *T. whipplei* infection. TLR2 has been shown to be overexpressed in intestinal lesions of Whipple's disease [24]. Recently, TLR2 and the intracellular receptor nucleotide-binding oligomerization domain 2 (Nod2) have been shown to cooperate in inducing the expression of FPR2 in microglial cells [31]. As FPR2 mediates the chemotactic activity of a variety of pathogen and host-derived peptides, it may actively participate in the macrophage infiltration observed in Whipple's disease lesions.

Second and more strikingly, we found that a robust type I IFN response was induced by viable *T. whipplei*. As compared with the plethora of reports delineating the critical role of type I IFN in host resistance to many types of viruses, only few papers report their involvement during bacterial infections [32]. Results from our study suggest that type I IFN is induced in a MyD88-/TRIF-dependent pathway and demonstrate that, comparable to classical type I IFN triggering signals [33], IRF3 signaling is activated following *T. whipplei* infection. Activation of the transcription factor IRF3 is likely to be dependent on TBK1. Indeed, TBK1 is required for the activation and nuclear translocation of IRF3 in mouse embryonic fibroblasts (MEF). Moreover, *Tbk1* ^−/−^ MEF show marked defects in type I IFNs, Cxcl10, and RANTES gene expression after infection with either Sendai or Newcastle disease viruses or after engagement of the TLR3 and TLR4 by double-stranded RNA and LPS, respectively [34]. To our knowledge, type I IFNs have never been associated with the induction of M2 polarization of macrophages and are rather seen as M1 effectors [35]. However, our results strongly support this new association as data presented here suggest a positive feedback loop involved in BMDM response to *T. whipplei*. First, STAT1 was phosphorylated on Y701, and this phosphorylation was absent when we used IFNAR1^−/−^ BMDM. It has been shown that Stat1 activation is achieved by phosphorylation on Y701 that is followed by nuclear accumulation. For full transcriptional activity, Stat1 is also phosphorylated on S727 [36]. As *T. whipplei* induced the expression of several IFN inducible genes, it is likely that STAT was also phosphorylated on S727. Second, we found that the transcription of these IFN inducible genes was abolished when macrophages lacking the type I IFN receptor were used.

Our microarray analysis did not reveal any genes related to proinflammatory activities of macrophages, suggesting that *T. whipplei* is a weak inducer of inflammatory responses. Even if *T. whipplei* triggered a weak IκBα degradation, RelA translocation in the nucleus was not detected, despite strong translocation when cells were treated with LPS. This may be due to the lack of sensitivity of the immunofluorescence assay since IκBα, the gene of which is under the control of NF-κB is resynthetized and reached initial values by 3 h. Consistent with the weak activation properties of *T. whipplei*, we were not able to detect early MAPK signaling in macrophages. However, MAPKs were activated more lately, after 24 h. The kinetics of MAPKs activation suggest that p38, ERK and JNK might be activated by a secondary signal, emanating from the initial *T. whipplei*-macrophage interaction. Indeed, type I IFN receptor engagement for example, has been shown to induce MAPKs [37],[38]. Nevertheless, we found that only JNK phosphorylation was absent in IFNAR1^−/−^ BMDM, while activation of p38 and ERK was similar to that observed in their wt counterparts.

Another interesting feature of the *T. whipplei* - macrophage interaction revealed by this study is the induction of apoptosis. Macrophage apoptosis is probably linked to bacterial replication. Indeed, cells that are able to eliminate *T. whipplei* such as monocytes do not undergo apoptosis [22]. These results are strengthened by the fact that circulating levels of apoptotic markers such as nucleosomes are increased in patients with active Whipple's disease [23]. Here, *T. whipplei* induced BMDM apoptosis with a maximal response 18 h post infection, while heat-killed bacteria were unable to induce apoptosis (data not shown). Induction of apoptosis appeared associated with i) type I IFN response and ii) JNK signaling. Apoptosis was inhibited by around 60% when IFNAR1^−/−^ BMDM were infected with *T. whipplei*. In the meantime, JNK activation was abrogated in these cells. By using a JNK-specific inhibitor, we were also able to inhibit by 50% *T. whipplei*-induced apoptosis. Hence, we can hypothesize that *T. whipplei* induces type I IFN, which binds its receptor, induces JNK phosphorylation to promote macrophage apoptosis. Recently, Jeon and colleagues have shown that type I IFNs activate a JNK-specific signaling cascade involving Rac1, MEKK1, MKK4 and leading to apoptosis through filamin B [39]. Type I IFNs have also been shown to activate JNK for the induction of apoptosis in some lymphoma cells [40]. Finally, type I IFNs also activate the caspase cascade leading to apoptosis [41]. However, we cannot rule out the hypothesis that macrophage apoptosis arise from other signals. Indeed, we found that genes encoding Fas (CD95/Apo1) and Tnfsf10 (TNF-related apoptosis-inducing ligand, TRAIL/Apo2L) were both significantly induced in BMDM in response to *T. whipplei*. Besides TNF itself, Fas and Tnfsf10 constitute two of the three death receptor/ligand systems that are responsible for the extrinsic induction of cell death [42]. Interestingly, Fas and Tnfsf10-dependent pathways involve JNK signaling and have been implicated in immunosuppressive and immunoregulatory functions [42],[43].

Besides its role on apoptosis induction, type I IFN appeared to be involved in replication of *T. whipplei*. Bacterial replication was partly inhibited in IFNAR1^−/−^ cells, as compared with wt BMDM. We also found that in wt BMDM, bacteria colocalized with Lamp1 but not with cathepsin D, as already described [44]. In contrast, in macrophage lacking the type I IFN receptor, bacteria mostly colocalized with cathepsin D. These results suggest that type I IFN can modulate, at least in part, microbial killing. Indeed, it has been shown that type I IFNs modulate vacuolar H^+^-ATPase-mediated acidification [45]. Interestingly, JNK activation was not required for *T. whipplei* replication and alteration of phagosome maturation. The role of JNK in phagosome conversion and bacterial killing is unclear as it seems to depend both on the upstream events (engaged receptor) and the pathogen itself. Indeed, it has been shown that JNK is involved in *Staphylococcus aureus* killing in a TLR2-dependent pathway through generation of superoxide, while its inhibition has no effect when cells are infected with *E. coli* [46]. From our study, two signals are emanating from the type I IFN receptor. The first involves JNK and leads to macrophage apoptosis while the second promotes alteration of phagosome maturation and bacterial replication independently of JNK. It has been shown that stimulation with type I IFN activates phosphatydilinositol-3 kinase (PI3K) and its downstream effectors [47]. As PI3K is involved in the modulation of phagosome maturation [48], it is therefore possible that PI3K activity is modulated by *T. whipplei* to alter its phagosome and to favour its replication. Further studies are needed to determine from where these two signals diverge.

A growing body of evidence shows that type I IFN participate in the host response to bacterial infection. However, their effects to the host can be either favorable or detrimental. For example, type I IFN response is critical in protecting the host against the extracellular pathogen group B Streptococcus [18]. In contrast, production of type I IFN during *L. monocytogenes* infection sensitizes macrophages to cell death [49]. Similarly, type I IFN production also appears detrimental for the host during infection with the *T. whipplei*-closely related *M. tuberculosis* [50]. *M. bovis* was shown to have enhanced replication rates in macrophages treated with type I IFN [51]. Our results suggest that the type I IFN induced by *T. whipplei* is detrimental for macrophages. Human infection with *T. whipplei* is a rare event despite the environmental ubiquity of the organism. Clinical features of Whipple's disease are non specific and it is clear that identifying the molecular mechanisms involved in type I IFN responses would have both clinical and therapeutic consequences.

## Materials and Methods

### Mice, cell culture and bacteria

BMDM from six week-old C57BL/6 and IFNAR1^−/−^ [52] mice were isolated as described previously [53]. Double MyD88/TRIF-deficient mice were bred from MyD88^−/−^ [54] and LPS2^−/−^ [55] mice. Mouse RAW 264.7 macrophages (American Type Culture Collection, ATCC N° TIB-71) were grown in Dulbecco's Modified Eagle Medium (DMEM) high glucose containing 10% FCS.

The strain Twist-Marseille of *T. whipplei* (CNCM I-2202) was cultured with HEL cells and purified as described previously [22]. Heat-killed *T. whipplei* was prepared by heating at 80°C for 1 h. All animal experiments followed the guiding principles of animal care and use defined by the Conseil Scientifique du Centre de Formation et de Recherche Experimental Médico-Chirurgical (CFREMC) and were approved by the ethics board of the university at which the experiments were performed (Faculté de Médecine de la Timone).

### Statistical analysis

All experiments were performed at least three times. One representative experiment is shown. Error bars represent SD of triplicate values from a representative experiment. *, P<0.05, Mann-Whitney's *U* test.

### Transient transfection

The eGFP-IRF3 plasmid was kindly provided by G. Querat (Marseille, France). RAW 264.7 macrophages were transfected with eGFP-IRF3 plasmid construct using Nucleofactor (Amaxa Biosystems), according to the manufacturer's recommendations.

IRF3-specific and control scramble siRNA were purchased from Santa Cruz Biotechnology. RAW 264.7 macrophages were transfected with IRF3-specific and control siRNA using Nucleofactor (Amaxa Biosystems), according to the manufacturer's recommendations.

### Macrophage infection and real-time quantitative PCR (qPCR)

*T. whipplei* organisms (MOI 50∶1) were added to BMDM for 4 h, washed to remove free bacteria and incubated for 12 days in RPMI 1640 containing 10% FCS and 2 mM glutamine. Every 3 days, macrophages were collected and DNA was extracted using the QIAamp DNA MiniKit (Qiagen). PCR was performed using the LightCycler-FastStart DNA Master SYBR Green system (Roche), as previously described [22].

### Immunofluorescence

Macrophages seeded on glass coverslips were infected with *T. whipplei* (MOI 50∶1) for 4 h, extensively washed to discard unbound bacteria and incubated in RPMI 1640 containing 10% FCS. At different time points, BMDM were fixed in 3% paraformaldehyde and permeabilized with 0.1% Triton X-100. Immunofluorescence labeling was performed according to standard procedures [56]. Briefly, BMDM were incubated with rabbit anti-*T. whipplei* (1∶2,000 dilution) antibodies (Ab) for 30 min [44] and rat anti-lamp1 (1∶1,000 dilution, clone 1D4B, purchased from DSHB) or rabbit anti-cathepsin D (1∶1,000 dilution, a gift from S. Kornfeld, Washington University School of Medicine, St. Louis, Missouri). Secondary Alexa Abs were purchased from Invitrogen and used at a 1∶500 dilution. Coverslips were mounted with Mowiol and examined by laser scanning microscopy using a confocal microscope (Leica TCS SP5) with a 63X/1.32-0.6 oil objective and an electronic Zoom 2X. Optical sections of fluorescent images were collected at 0.15-µm intervals using Leica Confocal Software and processed using Adobe Photoshop V7.0.1.

For the assessment of RelA (p65) translocation in the nucleus, the same procedure was followed except that BMDM were incubated with rabbit anti-p65 (RelA) monoclonal Ab (Cell Signaling).

### ELISA

Cell culture supernatants were assayed for IFN-β by ELISA (R&D Systems) according to the manufacturer's instructions.

### Microarray analysis

BMDM were infected with *T. whipplei* for 6 h (MOI 50∶1) and total RNA was extracted using the RNeasy minikit (Qiagen). The quality and the quantity of RNA preparation were assessed using the 2100 Bioanalyzer (Agilent Technologies). The 4X44k Mouse Whole Genome microarrays (Agilent Technologies) were used. Sample labeling and hybridization were performed according to the manufacturer recommendations (One-Color Microarray-Based Gene Expression Analysis). Briefly, 300 ng of total RNA and cyanine 3-labeled CTP were used to synthesize labeled cRNA using the Low RNA Input Fluorescent Amplification Kit (Agilent Technologies). Hybridizations were performed in triplicates for 17 h at 65°C using the *In situ* Hybridization Kit Plus (Agilent Technologies). Slides were scanned at 5 µm resolution with a G2505B DNA microarray scanner (Agilent Technologies). Image analysis and intra-array signal correction were performed using Agilent Feature Extractor Software 9.5.1.1. Global normalization by trimmed means was applied on raw datasets using Excel (Microsoft). Discrimination between samples was performed using the unpaired Student's *t* test. We only considered a gene as differentially expressed if the P value from Student's *t* test was below 0.01 and its absolute fold change was over 2.

To identify functional categories of genes that were over-represented in the data sets of modulated genes, we assigned Gene Ontology (GO) annotation by using the freely available online tools FatiGO Search (http://babelomics.bioinfo.cipf.es/) and DAVID Bioinformatics Resources 2008 (http://david.abcc.ncifcrf.gov/).

All transcriptional profile files have been submitted to the GEO database at NCBI (accession number GSE16180).

### Quantitative real-time RT-PCR

cDNA was synthesized from 1 µg of total RNA using SuperScript II RNase H reverse transcriptase (Invitrogen). Specific primers for each gene were designed using Primer3Plus, available online at http://www.bioinformatics.nl/cgi-bin/primer3plus/primer3plus.cgi. The sequences of the targeted genes are listed in Table 2. Quantitative RT-PCR was performed using LightCycler-*Fastart* DNA Master *SYBR Green* (Roche Diagnostics) and data acquired with the ABI PRISM 7900 HT (Applied Biosystems). Gene expression was normalized to the β-actin gene, relative expression of respective genes was calculated using comparative threshold cycle method [57].

**Table 2:** Primers used.

Target	Upstream primer (5′ → 3′)	Downstream primer (5′ → 3′)
*mmp14*	CCCAGATAAGCCCAAAAACC	GCATTGGGTATCCATCCATC
*irg1*	TTCCAAGCCTGGATTCTCAC	TCACAAGGTGCTTTCTCACG
*ifit2*	TACCCATCAGCAAGATGCAC	CTGTGTCAAAGCGCTCAAAG
*arg2*	TGGATCAAACCTTGCCTCTC	GCCGATCAAATGTCTGTTCC
*ifi44*	GCATATGCTGCATTGTCACC	TCCATTCCCAGTCCTTTCAG
*ccl22*	TCCCAGGGGAAGGAATAAAC	TTGTGGTCCCATATGCTGTC
*ifit1*	ATGGGAGAGAATGCTGATGG	CCCAATGGGTTCTTGATGTC
*olr1*	TGGCTATGGGAGAATGGAAC	CAGTTTTCAGCGAACACAGC
*nfkbie*	AAGGATTGCAGACGAGGAAG	ATCCCCATTTTCCAGGTAGC
*ccl17*	AAAGGGGCCATTCCTATCAG	CCAATCTGATGGCCTTCTTC
*mx2*	AGACAAAGCATGGCACTTCC	ACTGGATGATCAAGGGAACG
*mx1*	TGCTCATCTCCGACTGTTTG	TGGGGTACACAGGTGAAATG
*Ifnb1*	CCCTATGGAGATGACGGAGA	TCCCACGTCAATCTTTCCTC
*actb*	TGGAATCCTGTGGCATCCATGAAAC	TAAAACGCAGCTCAGTAACAGTCCG

These primers were designed using the primer3 tool available at the following website: http://frodo.wi.mit.edu/.

### Western blotting

Macrophages were stimulated with either *T. whipplei* (MOI 50∶1) or *Escherichia coli* 055∶B5 LPS (100 ng/ml, Sigma). At designated times, BMDM were washed with ice-cold PBS. Cells were then scrapped in ice-cold RIPA buffer (20 mM Tris-HCl, 200 mM NaCl, 1 mM EDTA, 1% Triton-X100, pH 7.5) containing protease inhibitor (Complete, Roche) and phosphatase inhibitor (Phosphostop, Roche) cocktails. The cell lysates were cleared by centrifugation at 14,000 rpm for 15 min at 4°C and stored at −80°C. Cell lysates were examined for equal amounts of protein by the Bradford method using γ globulin as a standard [58]. Samples were loaded onto 10% sodium dodecyl sulfate polyacrylamide gels, electrophoresed and transferred onto nitrocellulose membranes (Amersham). The membranes were blocked in PBS with 0.05% Tween 20 (PBST) supplemented with 3% powdered milk and then incubated with primary Abs against phospho-p38, total p38, phospho-ERK1/2, total ERK1/2, phospho-JNK, α-tubulin (Cell signaling), IκBα (Calbiochem) or IRF3 Ab (Santa Cruz) as indicated by manufacturers. The blots were washed with PBST and incubated with a secondary Ab, either horseradish peroxidase-conjugated anti-rabbit or anti-mouse immunoglobulin (Pierce) in PBST plus 3% powdered milk. The bound Abs were detected using Immobilon Western Chemiluminescent HRP substrate (Millipore).

### Apoptosis determination by Terminal transferase deoxytidyl uridine end labeling (TUNEL) staining

Detection of apoptosis by TUNEL was performed using *In Situ* Cell Death Detection Kit, TMR red (Roche) according to the manufacturer's instructions. JNK inhibition was performed using SP600125 (Sigma) at 50 µM for 30 min prior infection. As a control, apoptosis was induced by exposing cells to ultraviolet (UV) as described previously [59]. After treatment as indicated, cells on glass coverslips were fixed in 4% paraformaldehyde for 15 min, washed in PBS and permeabilized with 0.1% Triton-X100 in 0.1% sodium citrate for 2 min. Cells were then incubated with the TUNEL mixture containing TMR-dUTP and terminal deoxynucleotidyl transferase for 1 h. Cells were washed in PBS and nuclei were stained with DAPI before mounting with Mowiol. Positive controls were carried out by incubating cells with 3 U/ml DNase I prior labeling procedures. Negative controls were done by incubating cells with label solution (without terminal deoxynucleotidyl transferase). Apoptosis was quantified as follows. Coverslips were examined in fluorescence mode with a Leica microscope equipped with a Nikon digital camera using a 10X objective lens. Three to five fields per condition (100 to 300 cells each) were observed. The number of TUNEL-positive and DAPI-stained nuclei were determined and the apoptosis percentage was expressed as the ratio between TUNEL-positive and DAPI-stained nuclei ×100.

### List of LocusLink accession numbers for genes and proteins mentioned in the text

*arg2*, 11847; *ccl17*, 20295; *ccl22*, 20299; *clec4e*, 56619; *cxcl10*, 15945; *fas*, 14102; *fpr2*, 14289; *gbp2*, 14469; *ifi44*, 99899; *ifit1*, 15957; *ifit2*, 15958; *ifit3*, 15959; *ifnar1*, 15975; *ifnb1*, 15977; *il1rn*, 16181; *irf3*, 54131; *irg1*, 16365; *mx1*, 17857; *mx2*, 17858; *myd88*, 17874; *olr1*, 108078; *tlr2*, 24088; *tnfsf10*, 22035; *trif* (*ticam1*), 106759.

## Supporting Information

Figure S1 Time course of IFN-β expression and production in BMDM. (A) BMDM were stimulated with *T. whipplei* (MOI 50∶1) for the indicated time points and IFN-β expression was monitored using qRT-PCR. Results are expressed as the ratio of expression levels in stimulated cells vs. uninfected cells relative to β actin. (B) BMDM were stimulated with *T. whipplei* (MOI 50∶1) for the indicated time points and cell supernatants were assessed for IFN-β by ELISA (*n* = 3).
(0.27 MB PDF)

Figure S2 IRF3 knock-down efficiency. (A) RAW cells were transiently transfected with IRF3-specific siRNA (si-IRF3), control scramble siRNA (si-SCR) or left untransfected (control). After 24 h and 48 h, lysates were analyzed by immunoblotting. IRF3 blots were stripped and reprobed for tubulin as loading controls. Densitometry values of the IRF3 autoradiographs were normalized to tubulin. (B) RAW cells were transiently transfected with IRF3-specific siRNA (si-IRF3), control scramble siRNA (si-SCR) or left untransfected (control) 24 h before stimulation with *T. whipplei* (MOI 50∶1). After 6 h, cell supernatants were harvested and IFN-β production was assessed by ELISA (*n* = 3).
(0.56 MB PDF)

Figure S3 Bacterial replication proceeds independently of JNK activation. (A) BMDM were treated or not with the JNK specific inhibitor SP600125 for 30 min. Cells were then infected with *T. whipplei* (MOI 50∶1) for 4 h. SP600125 was added during the infection procedure. Levels of bacterial DNA copy number were determined by qPCR (*n* = 3). (B) At day 6 post infection and in the presence of SP600125, *T. whipplei* organisms, lamp-1 and cathepsin D were visualized by laser scanning microscopy.
(0.68 MB PDF)
