# Supplementary figures and images for: Type I Interferon Induction Is Detrimental during Infection with the Whipple's Disease Bacterium, Tropheryma whipplei
Source: PLoS Pathog. 2010 Jan 15;6(1):e1000722. doi: 10.1371/journal.ppat.1000722 (PMC2798751; doi:10.1371/journal.ppat.1000722)

**A**

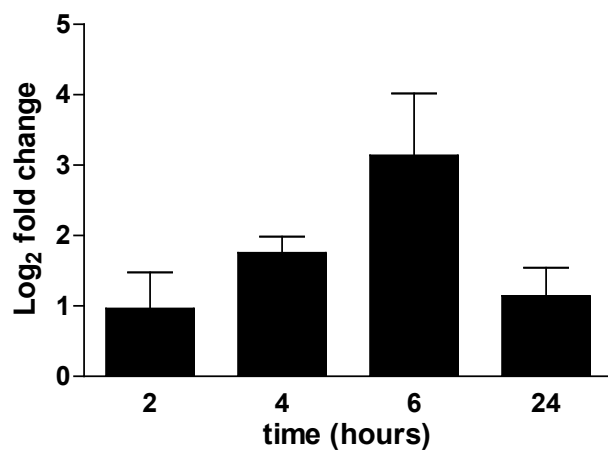

**B**

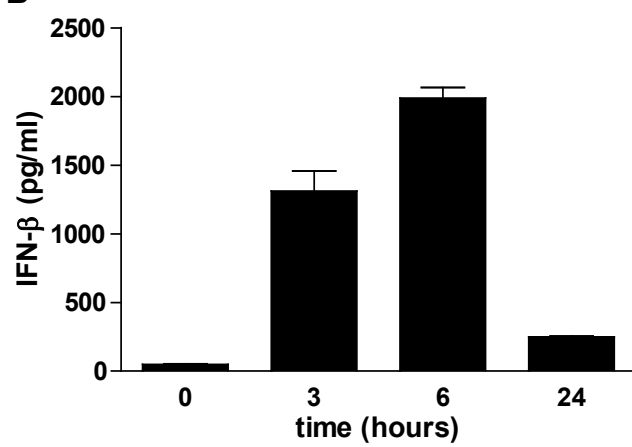

Supplement: Figure S1 — Time course of IFN-β expression and production in BMDM. (A) BMDM were stimulated with T. whipplei (MOI 50∶1) for the indicated time points and IFN-β expression was monitored using qRT-PCR. Results are expressed as the ratio of expression levels in stimulated cells vs. uninfected cells relative to β actin. (B) BMDM were stimulated with T. whipplei (MOI 50∶1) for the indicated time points and cell supernatants were assessed for IFN-β by ELISA (n = 3). (0.27 MB PDF) [file ppat.1000722.s001.pdf]

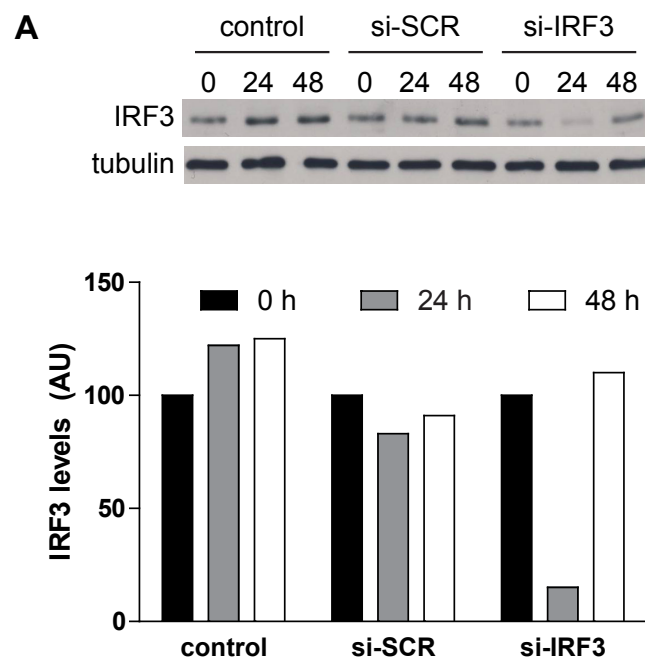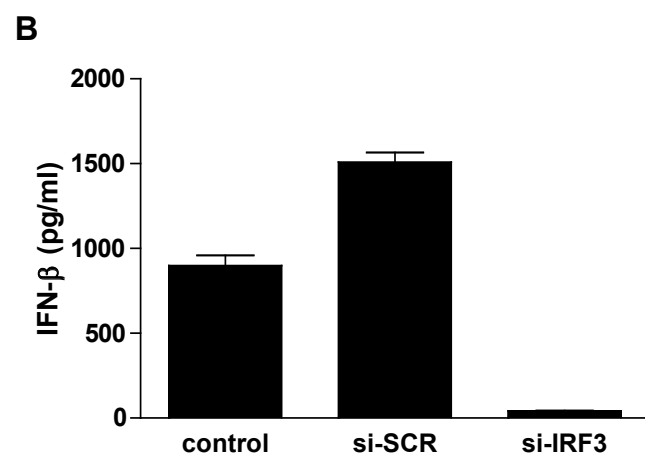

Supplement: Figure S2 — IRF3 knock-down efficiency. (A) RAW cells were transiently transfected with IRF3-specific siRNA (si-IRF3), control scramble siRNA (si-SCR) or left untransfected (control). After 24 h and 48 h, lysates were analyzed by immunoblotting. IRF3 blots were stripped and reprobed for tubulin as loading controls. Densitometry values of the IRF3 autoradiographs were normalized to tubulin. (B) RAW cells were transiently transfected with IRF3-specific siRNA (si-IRF3), control scramble siRNA (si-SCR) or left untransfected (control) 24 h before stimulation with T. whipplei (MOI 50∶1). After 6 h, cell supernatants were harvested and IFN-β production was assessed by ELISA (n = 3). (0.56 MB PDF) [file ppat.1000722.s002.pdf]

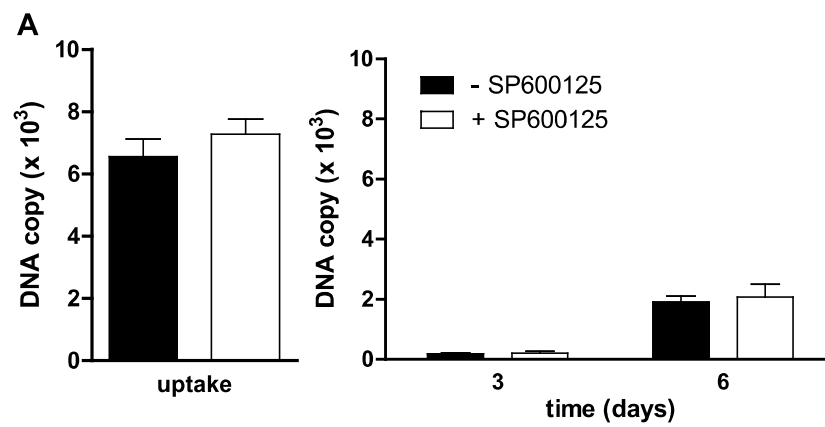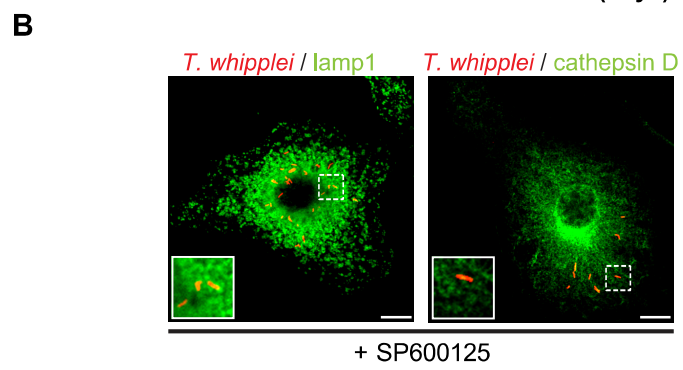

Supplement: Figure S3 — Bacterial replication proceeds independently of JNK activation. (A) BMDM were treated or not with the JNK specific inhibitor SP600125 for 30 min. Cells were then infected with T. whipplei (MOI 50∶1) for 4 h. SP600125 was added during the infection procedure. Levels of bacterial DNA copy number were determined by qPCR (n = 3). (B) At day 6 post infection and in the presence of SP600125, T. whipplei organisms, lamp-1 and cathepsin D were visualized by laser scanning microscopy. (0.68 MB PDF) [file ppat.1000722.s003.pdf]
